# Hearing siblings’ voices: exploring the (online) support needs of siblings of children with a chronic condition

**DOI:** 10.1186/s41687-019-0102-9

**Published:** 2019-02-12

**Authors:** M. M. H. Joosten, H. Maurice-Stam, L. Scholten, M. A. Grootenhuis

**Affiliations:** 1 Emma Children’s Hospital, Amsterdam UMC, University of Amsterdam, Psychosocial Department, Meibergdreef 9, Postbox 22660, 1100 DD Amsterdam, the Netherlands; 2 Princess Maxima Center for Pediatric Oncology, University Medical Center, Lundlaan 6, Postbox 85090, 3508 AB Utrecht, The Netherlands

**Keywords:** Siblings, Chronic condition, Online support needs, E-health, Participation

## Abstract

**Background:**

Siblings of children and adolescents with a chronic condition are at risk for developing psychosocial problems. It is important, that they receive appropriate support according to their needs. A sibling-specific module of an existing online intervention (*Op Koers Online)* for adolescents with a chronic condition might be an appropriate way to offer psychosocial support to siblings. The aim of the current study is to identify siblings’ online support needs in order to develop a sibling-specific module of the existing *Op Koers Online* intervention.

**Results:**

A total of 91 siblings (mean age 15.2 years, Standard Deviation 2.7) of children with a chronic condition completed an online questionnaire; nine semi-structured interviews were held additionally. Of all participants, 55% would like to initiate or increase contact with other siblings of children with a chronic condition and 46% of those were interested in an online chat course. The themes for online support considered most important were *impact on daily life, worrying about brother’s/sister’s future, handling other people’s reactions,* and *how attention is divided within the family*.

**Conclusions:**

Siblings are interested in peer contact and online support. *Op Koers Online* for siblings seems to be a suitable intervention to offer online psychosocial support. The next step is to develop a sibling specific module of the *Op Koers Online* course, taking into account the identified themes.

## Background

In the Netherlands, 15–20% of children live with a chronic condition [30]. It is estimated that this concerns around 500,000 children. Many siblings are also affected by chronic conditions during childhood. Children and adolescents growing up as a sibling of a child with a chronic condition, might have to cope with difficult situations. They can experience loneliness [16], worry about the prognosis of the ill child’s condition, and their brother’s/sister’s condition might affect family life and daily life [9]. Siblings also report receiving less attention from their parents and having lost the companionship of the ill child [24]. They are described as being the “forgotten children” [14].

To date, the literature remains inconclusive about the psychosocial functioning of siblings of children with a chronic condition. The mixed findings could probably be explained by diversity in outcome measures, chronic condition of the child and characteristics of the sibling under study, including how long the sibling has been living with the chronically ill child [10, 23, 33]. Two meta-analyses suggest siblings have worse psychological functioning (anxiety, depression) [23], more internalizing and externalizing problems, and fewer positive self-contributions than peers without a chronically ill brother/sister [33]. Some research reports lower scores on several indicators of well-being, even though effect sizes are usually small [4]. Other findings, however, suggest that siblings’ levels of depression and quality of life are similar to those of peers without a chronically ill sibling [10, 12, 32].

Even though the findings of different studies are contradictory, siblings of children with a chronic condition deserve attention. For a population as vulnerable as those siblings, it is important to have insight into their support needs. In several studies parents have been asked to report about sibling needs. Research shows that the agreement between proxy-reported outcomes and self-reported outcomes is sometimes low [8]. Hence, sibling participation is of great importance, as siblings are experts by experience. Participation can help better match the care to their needs, and a sibling perspective can add their own values to the values of professionals [27, 29]. In cancer populations, research was conducted on siblings’ (unmet) needs [18, 26] and how to screen them [13, 19]. In chronic-condition populations, however, needs of siblings have not been studied extensively. Research about support needs of siblings of special-needs families shows that most siblings do not know where they can get support and that there is not enough support available [17]. The need for a better insight into siblings’ support needs is stressed so that suitable interventions can be developed [25]. Interventions directed at siblings fit into the concept of family-centered care, where attention is given not only to the patient but to the well-being of the other family members too.

Specific interventions for siblings of children with chronic conditions are scarce, as are studies looking into the effectiveness of such interventions. A recent systematic review included 17 studies on the effects of psychosocial interventions for siblings [25]: only one study focused on chronic conditions (cystic fibrosis and heart disease), the others focused mainly on cancer and mental illnesses. Findings in this review suggest that interventions aimed at improving psychological outcomes had a positive effect on siblings of children and adolescents with a chronic condition. They lead to improved knowledge about the illness, and to better externalizing and internalizing behavior scores.

As far as we know, only one intervention for siblings has been examined in the Netherlands [11]. It concerned a support group developed at Emma Children’s Hospital/Amsterdam University Medical Centers (UMC) that was conceived for siblings of children with cancer. The goal was to improve siblings’ coping strategies and reduce anxiety. The group course consisted of five weekly sessions led by two psychologists. Changes in the family situation and emotions as a result of living with an ill brother or sister were discussed. The study with a pre-post design suggested that, on average, 16.5 months after their sibling received a cancer diagnosis, children experienced less anxiety shortly after participation in the support group.

This support group has become the basis of the face-to-face *Op Koers* program (in English: On Track). The program now consists of group courses for children, parents, and siblings, and has separate modules for cancer and chronic conditions. All group courses are based on cognitive behavioral therapy in order to prevent or reduce psychosocial problems. Cognitive behavioral therapy focuses on recognizing cognitive distortions and teaching coping skills [1]. Sharing experiences with fellow-patients is an important part of the intervention [20]. Effectiveness of the face-to-face *Op Koers* group course has been studied in chronically ill children with a randomized controlled trial. The group course had a positive effect on coping skills such as positive thinking, and on internalizing and externalizing problems [22].

To better fit into the current online world and to overcome logistic barriers, the *Op Koers* program was further developed into online *Op Koers* group courses. Online interventions that use cognitive behavioral techniques seem to have a positive effect on depressive and anxious symptoms and general distress in adults with a chronic condition [28]. Peer support is suggested to have a positive effect on attitudes, beliefs and perceptions [21]. The first online *Op Koers* group course was developed for childhood cancer survivors (CCS) [15]. After establishing preliminary feasibility for CCS, the module was adapted for adolescents with a chronic condition (ages 12–18) and parents of children with a chronic condition (ages 0–18) [3]. In these online courses, participants log on to a chat box at a set time for eight weekly sessions led by two psychologists. Participants are taught coping skills and share experiences with each other about themes that are related to themselves or their child having a chronic condition.

Although *Op Koers Online* is available for parents and children, there is no module yet for siblings of children with a chronic condition. An *Op Koers Online* group course, adapted for siblings (ages 12–18) might be an appropriate way to offer them psychosocial support. An online group course would allow siblings to get in contact with other siblings and share experiences on themes related to having a brother or sister with a chronic condition. It is not clear that such a group course meets siblings’ needs in terms of psychosocial support, though. We do not know whether siblings would like to have contact with peers through a chat course and what themes would be important to them. The aim of the current study is to identify siblings’ online support needs in order to develop a suitable cognitive-behavioral based chat course led by two psychologists: an *Op Koers Online* module for siblings.

## Methods

### Procedures & participants

An online questionnaire was developed to identify siblings’ online support needs. Additionally, in-depth information about online support needs was collected through semi-structured video-call interviews. In order to draw a large, heterogeneous sample of siblings of children with a wide variety of conditions, the questionnaire was published online with open access. Siblings were approached via patient associations’ websites, newsletters and social media, and flyers at the outpatient clinics of Emma Children’s Hospital/Amsterdam UMC. Information about the survey was also provided via announcements on websites and social media accounts linked to the psychosocial department of Emma Children’s Hospital/Amsterdam UMC.

Siblings could access the questionnaire via a link to the *Op Koers* website (www.opkoersonline.nl) between January and May 2017. Participants did not need a login code to complete the questionnaire. No names were used on the website – the data were stored and analyzed anonymously. Siblings were asked to leave their e-mail addresses only if they wanted to participate in a video call to further discuss their online support needs. Interviews were held between April and May 2017 and were audio-recorded.

Inclusion criteria for siblings were 1) to be 12–18 years old, 2) to have a brother or sister with a chronic condition, and 3) to be able to understand Dutch well enough to complete the questionnaires.

This study was conducted with permission of and in accordance with the regulations of the Medical Ethics Committee of Amsterdam UMC.

### Measures

#### Background characteristics

Background characteristics of participating siblings (age, sex, education) and their brothers or sisters with a chronic condition (diagnosis, age at disease onset, age) were collected with a self-developed questionnaire. The diagnoses were reported by the participating siblings and later categorized by the researcher with the assistance of a medical doctor at Emma Children’s Hospital/ Amsterdam UMC. When more than one diagnosis was reported, only the first one listed was taken into account. To gain insight into siblings’ psychosocial well-being as a background characteristic, information was gathered using the Dutch self-report version of the Strength and Difficulties Questionnaire (SDQ) [5–7]. Siblings were asked to rate 25 items (e.g. *Other people my age generally like me, I worry a lot*) on a three-point scale ranging from 0 *Not true* to 2 *Certainly true*. There are five scales (score range 0–10) consisting of five items, including emotional symptoms, conduct problems, hyperactivity/inattention, peer problems and prosocial behavior. A total difficulties score is calculated by adding the scores of all scales aside from the prosocial behavior scale. A higher score means more problems, except for prosocial behavior, where a higher score means more prosocial behavior. The internal consistencies of the total difficulty, emotional symptoms, conduct problems, hyperactivity/inattention, and prosocial behavioral scales were satisfactory, ranging from Cronbach’s α 0.5 to 0.8. Internal consistency of the peer problems scale was insufficient (α = 0.32). Therefore, this scale was not taken into account in further analyses. Mean scale scores in a Dutch population of boys and girls aged 11–16 were available [31], as well as cut-off scores with cut-off points chosen so that 80% of children scored normal, 10% borderline, and 10% abnormal [5].

#### Online support needs

Data on online support needs, and themes for online support were collected with a questionnaire, tailored for siblings of children with a chronic condition. The questionnaire was developed by the researchers, to identify whether siblings are interested in online peer support, with whom, in what form and discussing what themes. The themes included in the questionnaire were identified by clinical psychologists from the Psychosocial department of Emma Children’s Hospital/Amsterdam UMC, and based on existing literature and clinical experience. The questionnaire consisted of nine items, partly open (e.g. *What themes would you like discuss in online support*) and partly multiple-choice (e.g. *In what form would you like online support*). All multiple-choice questions are listed in Table 2.

In addition to administering the questionnaire, semi-structured interviews were conducted. Every interview was held with a fixed sequence of topics, to check for potential missed needs in the questionnaire. The topics were: 1) *Do you talk to others about your brother’s or sister’s condition, and would you like support?* 2) *What themes do you find important concerning your chronically ill brother or sister*? 3) *What should an online intervention look like?* Within these topics, siblings were free to talk about anything they found important.

### Data analysis

All analyses were conducted using The Statistical Package for Social Sciences 24 (SPSS Inc., Chicago). Descriptive analyses were performed on background characteristics and online support needs. Siblings’ mean SDQ scale scores were compared with weighted (by gender) mean scores from the Dutch norm population, using a one-sample t-test. Effect sizes (d) were calculated for the differences in mean scores between the siblings and the norm group, dividing the difference by the standard deviation in the norm group. Effect sizes (d) of up to 0.2 were considered to be small, effect sizes around 0.5 medium and effect sizes around 0.8 large [2]. In addition, binomial tests were performed to assess whether the percentage of siblings with scores in the abnormal or borderline range differed from the percentage (20%) with equivalent scores in the Dutch population.

To explore whether any themes in the online-support-needs questionnaire were missed, the audio tapes of the interviews were listened to.

## Results

### Participants

A total of 104 siblings of a child with a chronic condition completed the online questionnaire in the broad age range of 4–35 years, even though the provided information about the survey mentioned the eligible age range of 12–18 years. The researchers then decided to widen the eligible age range to 11–21 years so they could take data of more participants into account. A total of 91 participants fit that range (including six 11-year-olds and ≥ 19-year-olds).

Twenty-three siblings left their e-mail address in order to be contacted for a video call. Fourteen of them did not participate in an interview, due to either non-response at follow-up or planning difficulties. Nine interviews were held.

### Background characteristics

Background variables of participants are shown in Table 1. Of the 91 siblings, 61 (67.0%) were female. Mean age of participating siblings was 15.2 years with a Standard Deviation (SD) of 2.7. Mean age of their brother or sister with a chronic condition was 13.8 (SD 4.4) years. As the variety of medical diagnoses was large, only the most frequently reported diagnoses are presented in Table 1.

**Table 1:** Background characteristics

	%	Mean (SD)			
Participating siblings (*n* = 91)					
Age in years		15.2 (2.7)			
Sex (Female)	67				
Current education					
primary school	9.9				
secondary school	65.9				
advanced education	18.7				
None anymore	5.5				
* Strengths and difficulties (SDQ)* ^a^	Siblings (*n* = 91)			Norm^b^ (*n* = 1353)	
	Borderline/ Abnormal (%)	Total f Borderline/ Abnormal (%)	Mean (SD)	Weighted mean (SD)	Effect size
Emotional symptoms	7.7 / 22.0	29.7*	3.5 (2.6)	2.4 (1.9)	0.58**
Conduct problems	4.4 / 6.6	11.0*	1.8 (1.5)	1.9 (1.5)	−0.06
Hyperactivity/inattention	12.1 / 28.6	40.7***	4.7 (2.7)	3.8 (2.3)	0.37**
Prosocial behavior	3.3 / 5.5	8.8**	8.2 (1.8)	7.3 (2.3)	0.41**
Total difficulties	15.4 / 19.8	35.2**	11.9 (5.9)	10.0 (4.9)	0.39**
Child with chronic condition	%	Mean (SD)			
Age child in years (*n* = 90)		13.8 (4.4)			
Age at disease onset (*n* = 91)					
during or shortly after birth	46.2				
0–5 years old	27.5				
6–12 years old	19.8				
13–18 years old	6.6				
First named diagnosis					
neurological disorder	12.1				
cardiovascular disorder	11.0				
cystic fibrosis	11.0				
chromosomal/syndromal disorder	8.8				
metabolic disorder	8.8				
connective tissue disorder	7.7				
disorder in the locomotor system	6.6				
Crohn’s disease	6.6				
other	27.5				

*SD* standard deviation

^a^Higher scores indicate more problems, except for the prosocial behavior subscale

^b^Norm population is 11–16 years old. Sample participants age range: 11–21. Analyzing only the 11–16 year-olds did not yield different results

*p < 0.05, **p < 0.01, ***p < 0.001: siblings differ significantly from the norm

Regarding psychosocial well-being, Table 1 shows the percentage of siblings scoring in the *borderline/abnormal range* of the scores on the different scales and total difficulty scale of the SDQ: borderline 3.3–15.4%, abnormal 5.5–28.6%. The percentages of siblings with abnormal or borderline scores were significantly higher than the 20% in the Dutch population on the emotional symptoms, hyperactivity/attention and total difficulties scales. Compared to the Dutch weighted norm, siblings in our sample also had significantly higher mean scores on these scales, indicating more problems. Regarding conduct problems, the percentage of siblings with abnormal or borderline scores was significantly lower than the 20% in the Dutch population, while their mean scores did not differ from the Dutch population. Siblings also had higher scores on prosocial behavior (mean as well as percentage) than the Dutch population, indicating more prosocial behavior.

### Online support needs

Table 2 presents results on multiple-choice questions on online support needs and important themes for an online group intervention. In our sample, 39.6% of siblings does have contact with other siblings, mostly through friends and/or family (25.3% of the total sample). In addition, 55% answered *yes* or *maybe* to the question of whether they would like to initiate or increase contact with other siblings of children with chronic conditions. These 55% in turn were asked additional questions about who they would like to get in contact with and how they would prefer online support. This is described next.

**Table 2:**
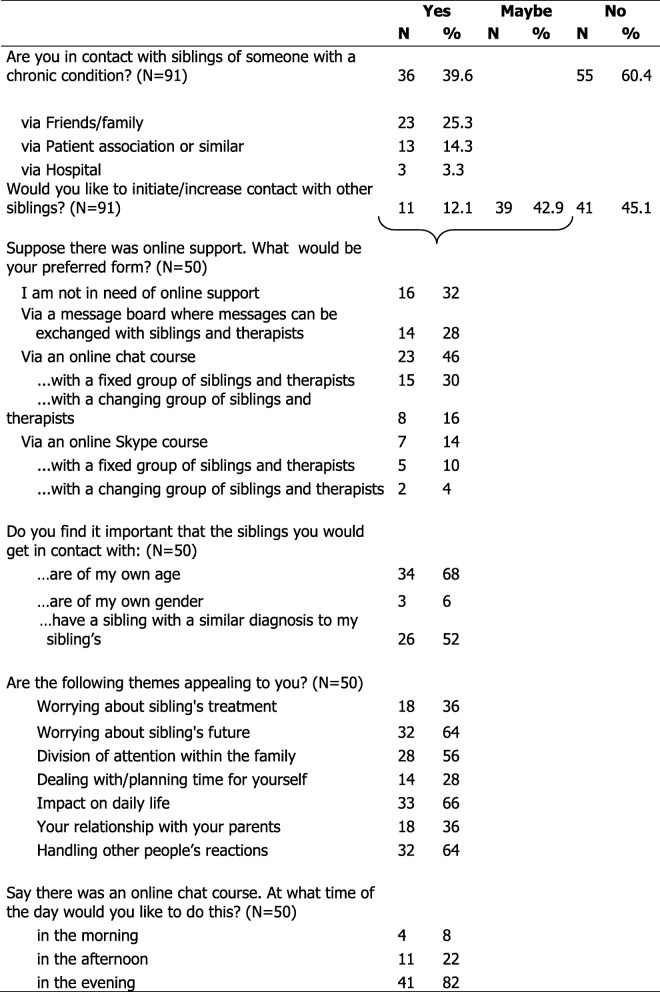
Results of multiple-choice questions about online support needs and themes for online support

Siblings were asked what characteristics they would find important in other siblings for online support. Comparable age (68%) and similar diagnosis of the brother/sister (52%) were regarded important for group meetings. Gender was regarded as less important – only 6% indicated preferring to get in contact with siblings of their own gender. Siblings were then invited to share their thoughts about how they would like to get in contact with other siblings, by asking them an open question. Key words that came up from at least 10% of respondents were *through an activity* (39%)*,* via *the internet* (24%)*, just have a talk and share experiences* (18%), and *casual, laid back* (14%)*.*

For modes of online support, 32% reported not being in need of any form of online support at all; 28% reported that they would like an online message board, 46% an online chat course, and 14% an online Skype course. Concerning time of the day that they would like an online intervention, 82% of participants answered *in the evening*. When asked about important themes for an online intervention, respondents were initially invited by open question to name the themes that are most important to them. A wide variety of answers were given, the most common themes being *how others cope with the situation in general* (28%) and *parent’s attention *(10%). Next, participants were asked to indicate whether a set of listed themes appealed to them. All themes (see Table 2) were considered appealing by at least one third of respondents. Four out of seven themes were considered appealing by more than half of respondents: *impact on daily life* (66%), *worrying about brother’s/sister’s future* (64%), *handling other people’s reactions* (64%) and *how attention is divided within the family *(56%).

The interview data revealed no additional important themes for online support for siblings. Just as in the survey data, some of the siblings stated they were not in need of support, as they weren’t experiencing many difficulties growing up with a ill brother or sister. Most siblings however, did elaborate on the different themes that were already listed in the questionnaire, and were very willing to share their thoughts and feelings with the interviewer. They appreciated the sincere attention the interviewer gave to their experiences, feelings, and needs.

## Discussion

The aim of this study was to identify siblings’ online support needs in order to develop an *Op Koers Online* intervention for siblings. The needs were assessed with a customized questionnaire supplemented with semi-structured interviews.

Because siblings might have to cope with difficult situations, they might experience psychosocial problems. Hence, psychosocial well-being of participants was assessed as a background characteristic. In our sample, psychosocial well-being of siblings was worse than the norm, except for conduct problems. Siblings also reported more prosocial behavior than the norm. This is in line with what is experienced in clinical practice; siblings of children with chronic conditions tend to be typically friendly and helpful. These results stress the vulnerability of the siblings in our sample. The finding that siblings had more psychosocial problems than peers in some domains but not in all is also in line with previous research [4, 32]. Effect sizes in the present study were moderate, whereas former studies show mostly small effect sizes [4]. The current study provides no information about possible positive effects of being a sibling of a child with a chronic condition. Better understanding of positive effects could provide insight into potential helpful coping strategies that others can benefit from.

An indication of the feasibility of a peer support intervention is given by the result showing that 55% of participants might like to initiate or increase contact with other siblings. This corresponds with findings of a needs assessment conducted by the Netherlands Youth Institute [17].

More in detail, most siblings would like to get in touch with siblings of about the same age, regardless of gender, which is in line with the current design of the *Op Koers Online* course for children with a chronic condition. This course is aimed at boys and girls of secondary school age. Half (52%) of the participants stated that they find it important that the other siblings they get in touch with have a brother or sister with a similar condition. The current *Op Koers Online* interventions for parents and adolescents with a chronic condition focus on the similarities between children with different chronic conditions, rather than on the differences between diagnosis groups. It is believed that even though diagnoses may differ, the psychosocial challenges that come with having a chronic condition are mostly the same [20]. A pilot study on *Op Koers Online* groups with heterogeneous diagnoses among participants or children from participants shows promising results (Douma et al. 2019). It is plausible that the heterogeneity of the groups will also work for siblings. Furthermore, heterogeneous groups give siblings of children with rare illnesses the opportunity to participate in a group intervention. It is therefore important that, before introducing *Op Koers Online* to siblings, psychologists make sure to explain them that having a sibling with a chronic condition has generic consequences for different diagnoses.

Our results additionally indicate that an online intervention fits into the digital environment adolescents live in. Of the participants that stated they would like to get in touch with other siblings, 46% would like to receive online support via an online chat course.

Another interesting result was that siblings appeared to prefer an evening rather than a daytime. So far, *Op Koers Online* courses for adolescents with a chronic condition mostly take place during daytime. Participation rates might be enhanced if the point of time of the group course fits the adolescents’ schedule, thus asking flexibility of the team providing the courses.

On the topic of important themes, the open question yielded a wide variety of answers. The most common answer was (a variant on) *how others cope with the situation in general* (28%). This suggests that participants were mostly interested in peer contact in general. All pre-listed themes in the multiple-choice question were considered important by at least 30% of participants. It is important to take these themes into account when further developing an intervention for siblings.

The finding that the semi-structured interviews yielded no new information suggests that the questionnaire was appropriate for gaining insight into siblings’ online support needs and important themes. The interviews did stress the importance of an intervention for siblings, since most of them reported having trouble with one or more aspects of growing up with a chronically ill brother or sister. It is also important to take into account that some siblings stressed that peer contact should be *casual, laid back* or *just having a talk.* This shows that, even though siblings might experience difficulties, they would not like to participate in a group course with too intense of a focus. Finding a balance between giving enough attention to the sibling’s difficulties and also “keeping it light enough” are of great importance in developing a course for siblings.

One must keep in mind that this study focuses on online support and provides no insight into support needs other than online ones. The 32% of participants that indicated not being interested in online support may have other support needs. Awareness for siblings’ needs is of the utmost importance in pediatrics.

This study had some limitations. The first one is about the sample. The open recruitment strategy had the advantage of being able to include more participants. However, as a consequence of this no information about response rates or differences between non-respondents and respondents is available. In other words, we do not know whether the results are representative for all siblings of children with chronic conditions. One might argue that siblings with more support needs were more likely to complete the questionnaire. This could have led to an overestimation of online support needs and possibly explains why the psychosocial wellbeing of the siblings in our study appeared to be worse than that found in previous studies. Also, two-thirds of the participants were sisters and all the semi-structured interviews were held with girls, since no boys signed up. Overrepresentation of girls is not uncommon in questionnaire studies. Girls internalize more than boys, so expectedly they are more likely to search for support for their emotional problems. The findings should therefore be interpreted with caution.

## Conclusions

All results taken into account, an *Op Koers Online* module for siblings seems to be a suitable intervention for part of the sibling population. Siblings appeared to be interested in peer contact and online support. The next step is to develop a sibling-specific module for the *Op Koers Online* course, taking into account the identified themes. The sibling participation of our study contributed to our process of developing the online course program. Once the intervention is developed, further research should focus on feasibility and effectiveness of *Op Koers Online* for siblings.

## Abbreviations

Amsterdam UMC: Amsterdam University Medical Centers
SD: Standard Deviation
